# Integrated Histological and Transcriptomic Analyses Reveal Distinct Structural and Molecular Features of the Calipash and Muscle in the Chinese Soft-Shelled Turtle (*Pelodiscus sinensis*)

**DOI:** 10.3390/ani16182960

**Published:** 2026-09-20

**Authors:** Yi-Ming Xie, Li-Ming Xiong, Yi-Ming Xiang, Xiang Zhao, He-Wei Xiao, Ya-Zhou Hu, Xiao-Qing Wang, Shu-Ting Xiong, Qin Qin

**Affiliations:** 1 Fisheries College, Hunan Agricultural University, Changsha 410128, China; yiming.xie@stu.hunau.edu.cn (Y.-M.X.);; 2 Yuelushan Lab, Changsha 410128, China; 3 Hunan Fisheries Research Institute and Aquatic Products Seed Stock Station, Hunan Academy of Agricultural Sciences, Changsha 410153, China

**Keywords:** *Pelodiscus sinensis*, calipash, muscle, collagen, extracellular matrix (ECM), transcriptomics

## Abstract

Calipash and muscle are the two most economically important edible tissues in Chinese soft-shelled turtle (*Pelodiscus sinensis*), However, the molecular and structural basis underlying their tissue-specific characteristics remains insufficiently understood. In this study, histological and protein-level analyses revealed that the calipash exhibits a highly organized multilayered architecture, in contrast to the myofiber-dominated organization of muscle. Type I collagen was highly abundant throughout the calipash and showed a spatially heterogeneous distribution, with particularly dense deposition in the outer layers and a mixture of type I and type III collagens in the innermost stromal layer. In contrast, collagen in the muscle was mainly restricted to the interstitial spaces and was predominantly composed of type I collagen, with relatively limited type III collagen. Using calipash as the reference group, transcriptome sequencing identified 6916 differentially expressed genes (DEGs) between calipash and muscle, including 3453 genes upregulated and 3463 genes downregulated in calipash. Calipash-specific genes were predominantly enriched in epidermal development, keratinocyte differentiation, ECM organization, and immune-related pathways, consistent with its specialized skin-like architecture and collagen-rich ECM. In contrast, muscle-specific genes were mainly associated with oxidative phosphorylation, mitochondrial function, myofibril assembly, and muscle contraction, in line with its mitochondria-enriched contractile architecture and energy-demanding characteristics. Focusing on collagen gene families, we found that *col1a1/2* exhibited the highest transcriptional abundance in both tissues. Notably, qPCR and WB confirmed that this transcriptional advantage translated into substantial type I collagen protein deposition. These findings provide new insights into the molecular basis of tissue-specific texture and the biological differences between calipash and muscle in *P. sinensis*.

## Simple Summary

The calipash and muscle are important edible and economic tissues of the Chinese soft-shelled turtle, but they differ markedly in texture. By comparing their histological structures and molecular profiles, we discovered that the calipash is a highly specialized skin-derived tissue. It is packed with a dense, highly organized network of type I collagen, which gives it its unique gelatinous characteristics. Conversely, the muscle is primarily responsible for energy metabolism, consisting mostly of muscle fibers. This study uncovers evidence of several biological mechanisms behind the distinct textures of these two important tissues.

## 1. Introduction

The Chinese soft-shelled turtle (*Pelodiscus sinensis*) is widely distributed in China and Southeast Asia, representing one of the most economically valued freshwater specialty aquaculture species [1]. In addition to its nutritional value, various tissues and products derived from *P. sinensis* have a long history of use in traditional Chinese medicine [2,3]. Among its edible tissues, calipash and muscle are highly favored by consumers [4]. Calipash, located at the margin of the carapace, is renowned for its gelatinous texture, smooth mouthfeel, and rich collagen content [5,6]. In contrast, muscle presents a typical fibrous and contractile texture [5,7]. Previous studies have investigated various biological and economically relevant traits of *P. sinensis*, including growth, embryonic development, pigmentation, and immune responses [8,9,10,11,12,13]. In particular, biochemical studies have characterized type I collagen from the calipash of *P. sinensis* [8]. In addition, recent transcriptomic and proteomic studies have implicated the PI3K-Akt and TGF-beta pathways in embryonic calipash development [10], and metabolome and transcriptome analyses have revealed molecular and metabolic characteristics of muscle, including changes in collagen-related genes [11]. However, the structural basis and molecular mechanisms underlying their distinct tissue architectures and textural properties remain largely unexplored.

Extracellular matrix (ECM) composition and organization are important determinants of tissue architecture and mechanical properties. Collagens constitute the major structural proteins of the ECM and form tissue-specific fibrillar and non-fibrillar networks that provide tensile strength, structural integrity, and mechanical support [14,15,16]. In particular, fibrillar collagens such as type I and type III collagen contribute to the formation and organization of dense connective tissues, whereas other collagen subtypes have specialized functions in epithelial basement membranes, cell–matrix interactions, and tissue-specific ECM structures [17,18,19]. Beyond collagen abundance, the spatial organization, fiber architecture, subtype composition, and degree of collagen cross-linking collectively influence ECM stiffness, elasticity, and resistance to deformation [17,18]. Collagen cross-linking stabilizes collagen fibrils and increases the mechanical strength and structural stability of connective tissues, whereas differences in collagen organization and maturation can contribute to distinct tissue mechanical properties. Therefore, differences in collagen deposition, composition, organization, and maturation may provide an important structural basis for the pronounced textural differences between calipash and muscle.

Despite the recognized importance of ECM composition in determining tissue structure and mechanical properties, the microstructural organization and molecular characteristics of calipash compared with muscle in *P. sinensis* remain poorly understood, particularly with respect to collagen composition and its associated regulatory networks. Thus, histological staining, including hematoxylin–eosin (H&E), Masson’s trichrome, and Sirius Red staining, was used to characterize tissue architecture and collagen distribution [20,21,22,23]. Collagen protein analyses and RNA sequencing (RNA-seq), which has been widely applied to investigate the molecular basis of economically important traits in aquaculture species [24,25,26,27,28], were further employed to characterize collagen features and identify differentially expressed genes (DEGs) and biological pathways associated with tissue-specific characteristics in *P. sinensis*.

## 2. Materials and Methods

### 2.1. Experimental Animals and Tissue Sample Collection

Nine healthy two-year-old Chinese soft-shelled turtles were obtained from the Hunan Fisheries Research Institute and Aquatic Products Seed Stock Station (Changsha, Hunan Province, China). During sampling, the animals were euthanized by tricaine methanesulfonate (MS-222) followed by cervical dislocation. The nine turtles were divided into three biological replicates, with tissues from three turtles pooled for each replicate. Calipash and hindlimb muscle tissues were pooled separately and divided into three aliquots: one was fixed in 4% paraformaldehyde for histological analysis, and the other two were rapidly frozen in liquid nitrogen and stored at −80 °C for RNA and protein extraction, respectively. All animal procedures were approved by the Animal Care Committee of Hunan Agricultural University, China (Approval No. 20241120).

### 2.2. Histological Analysis

After fixation in 4% paraformaldehyde for 24 h, calipash and muscle tissues were dehydrated through a graded ethanol series, cleared in xylene, infiltrated with paraffin for 3 h, embedded in paraffin blocks, and sectioned at 4 μm thickness. Sections were floated on a 42 °C water bath, mounted onto slides, and air-dried. After rehydration through a graded ethanol series, sections were stained with hematoxylin and eosin (H&E), Masson’s trichrome, and picrosirius red using commercial kits (Servicebio Technology Co., Ltd., Wuhan, China) according to the manufacturer’s instructions. Stained sections were dehydrated, cleared, and mounted with neutral resin. Images were captured using a light microscope and a polarized light microscope. For collagen area quantification, in each individual, three randomly selected microscopic fields were analyzed, and their mean value was used as one biological replicate. Thus, quantitative data were obtained from three individual animals (*n* = 3) per tissue, based on three fields per animal. Quantification and analysis were performed using Fiji (ImageJ, version 2.9.0/1.54f). Collagen-positive areas were quantified using Fiji (ImageJ) with the Color Threshold tool. The Hue, Saturation, and Brightness (HSB) ranges were set as follows: Hue: 180–255 (for blue-stained collagen in Masson’s trichrome), Saturation: 0–255, Brightness: 0–255. Thresholds were applied uniformly across all images, and the area fraction (%) was calculated as (collagen-positive area/total tissue area) × 100.

### 2.3. Immunofluorescence

Paraffin-embedded tissue sections were subjected to antigen retrieval in citrate antigen retrieval solution (Baiqiandu Biotechnology Co., Ltd., Wuhan, China) using a pressure cooker for 2 min. Endogenous peroxidase activity was blocked with 3% hydrogen peroxide. After blocking with 10% goat serum (Boster Biological Technology Co., Ltd., Wuhan, China) for 30 min, the sections were incubated with a rabbit anti-COL1A1 primary antibody (ABclonal Biotechnology Co., Ltd., Wuhan, China; 1:200 dilution) overnight at 4 °C. Subsequently, the calipash was incubated with FITC-conjugated goat anti-rabbit IgG and the muscle tissue with Cy3-conjugated goat anti-rabbit IgG (SeraCare Life Sciences, Inc., Milford, MA, USA) for 1 h at room temperature in the dark, thereby establishing an indirect immunofluorescence assay (IFA). Tyramide signal amplification was performed using TSA reagent (Baiqiandu Biotechnology, Wuhan, China). Nuclei were counterstained with DAPI (1 μg/mL) for 10 min. Sections were mounted and imaged under a fluorescence microscope.

### 2.4. Western Blot Analysis

Total protein was extracted from calipash and muscle. For each biological replicate, 500 mg of pooled tissue from three individuals was homogenized in 1 mL RIPA lysis buffer containing protease inhibitors (Epizyme Biomedical Technology Co., Ltd., Shanghai, China). Protein concentrations were determined using a BCA protein assay kit (Epizyme Biomedical Technology). Equal amounts of protein (30 μg per lane) were separated by 8% SDS-PAGE and transferred onto PVDF membranes. Biological replicates (*n* = 3 pools) were analyzed independently. After blocking with 5% non-fat milk for 2 h at room temperature, the membranes were cut horizontally according to the molecular weights of target proteins. The upper portion was incubated overnight at 4 °C with a rabbit anti-COL1A1 primary antibody (1:2000 dilution; ABclonal Biotechnology), and the lower portion was incubated with a rabbit anti-β-actin antibody (1:2000 dilution; ABclonal Biotechnology) as a loading control. Following washes with TBST, the membrane strips were incubated with an HRP-conjugated secondary antibody for 2 h at room temperature. After further washes, protein bands were visualized using an enhanced chemiluminescence (ECL) kit (Epizyme Biomedical Technology). Chemiluminescence signals were captured with exposure times ranging from 30 s to 3 min to ensure optimal signal detection without saturation.

### 2.5. RNA Extraction, Library Construction, and Sequencing

Total RNA was isolated using TRIzol reagent (TransGen Biotech, Beijing, China). RNA was stored at −80 °C. High-quality samples (OD260/280 ≥ 1.9, OD260/230 ≥ 1.8) were used for library construction. A total of 3 μg of RNA per pooled sample was used to construct RNA libraries with the VAHTS Universal V10 RNA-seq Library Prep Kit (Vazyme Biotech Co., Ltd., Nanjing, China), following the manufacturer’s protocol. The procedure included polyA-selected RNA extraction, RNA fragmentation, random hexamer-primed reverse transcription, and paired-end (2 × 100 bp) sequencing on a DNBSEQ-T7 platform by Igenebook Biotechnology Co., Ltd. (Wuhan, China). All raw sequencing data generated in this study have been deposited in the NCBI BioProject database with accession number PRJNA1332075.

### 2.6. Transcriptome Data Processing and Identification of Differentially Expressed Genes (DEGs)

Low-quality reads were filtered using fastp v0.21.0, and clean reads were mapped to the reference genome [GCE 049634645.1] using HISAT2 v2.1.0. Transcript abundance was quantified using featureCounts v1.6.0. Raw read counts were used for differential expression analysis with edgeR v3.36.0, while fragments per kilobase of transcript per million mapped fragments (FPKM) were calculated to represent normalized transcript expression levels [29]. Differentially expressed genes (DEGs) were identified with thresholds of false discovery rate (FDR) < 0.05 and |log_2_ fold change| ≥ 1 [30].

### 2.7. Functional Enrichment and Identification of Tissue-Specific Genes

To characterize the transcriptional divergence between the two tissues, we performed an overlapping analysis (Venn diagram analysis) based on the expressed genes. Genes with a mean FPKM ≥ 5 across biological replicates were considered reliably expressed, following a conservative expression threshold used in previous fish transcriptome studies [31]. Thus, calipash-specific genes were significantly expressed (FPKM ≥ 5) exclusively in calipash but not in muscle (FPKM < 5) in the Venn analysis. Subsequently, the identified tissue-specific gene sets were subjected to Gene Ontology (GO) and Kyoto Encyclopedia of Genes and Genomes (KEGG) pathway enrichment analyses to investigate their potential biological roles and signaling pathways relevant to tissue identity. The significance of enrichment was assessed using a hypergeometric test, and the resulting *p*-values were adjusted for multiple testing using the Benjamini–Hochberg FDR correction. GO terms and KEGG pathways with an adjusted *p*-value ≤ 0.05 were considered significantly enriched [32].

### 2.8. Validation by Real-Time Quantitative PCR (RT-qPCR)

For each cDNA sample, total RNA from three animals was pooled, and cDNA was synthesized using the RevertAid First Strand cDNA Synthesis Kit (Thermo Fisher Scientific, Waltham, MA, USA) according to the instructions. RT-qPCR was performed on a CFX96 Touch Real-Time PCR Detection System (Bio-Rad Laboratories, Hercules, CA, USA) using ChamQ Universal SYBR qPCR Master Mix (Vazyme Biotech Co., Ltd., Nanjing, China). Each reaction was performed in a total volume of 10 μL, and contained 2× Master Mix, specific primers and cDNA template, following the manufacturer’s protocol. The thermal cycling protocol consisted of an initial denaturation step, followed by 40 cycles of two-step amplification (denaturation and annealing/extension), and then a melting curve analysis. Relative gene expression was calculated using the 2^−ΔΔCt^ method [33], normalized to the internal reference gene, β-actin. Melt curve analysis confirmed a single peak for each amplicon, verifying primer specificity. All primers used for the qPCR assays are listed in Table A1.

### 2.9. Data Collection and Statistical Analysis

Data are presented as mean ± standard deviation (SD). Graphs were generated using GraphPad Prism 6 (GraphPad Software, Boston, MA, USA). Statistical significance was assessed by a two-tailed paired Student’s *t*-test using SPSS (IBM SPSS Statistics, version 32.0.0) software. Significant differences (* *p* < 0.05) between groups are indicated by distinct lowercase letters and asterisks.

## 3. Results

### 3.1. Comparative Histological Characterization Between Calipash and Muscle

**Figure 1:**
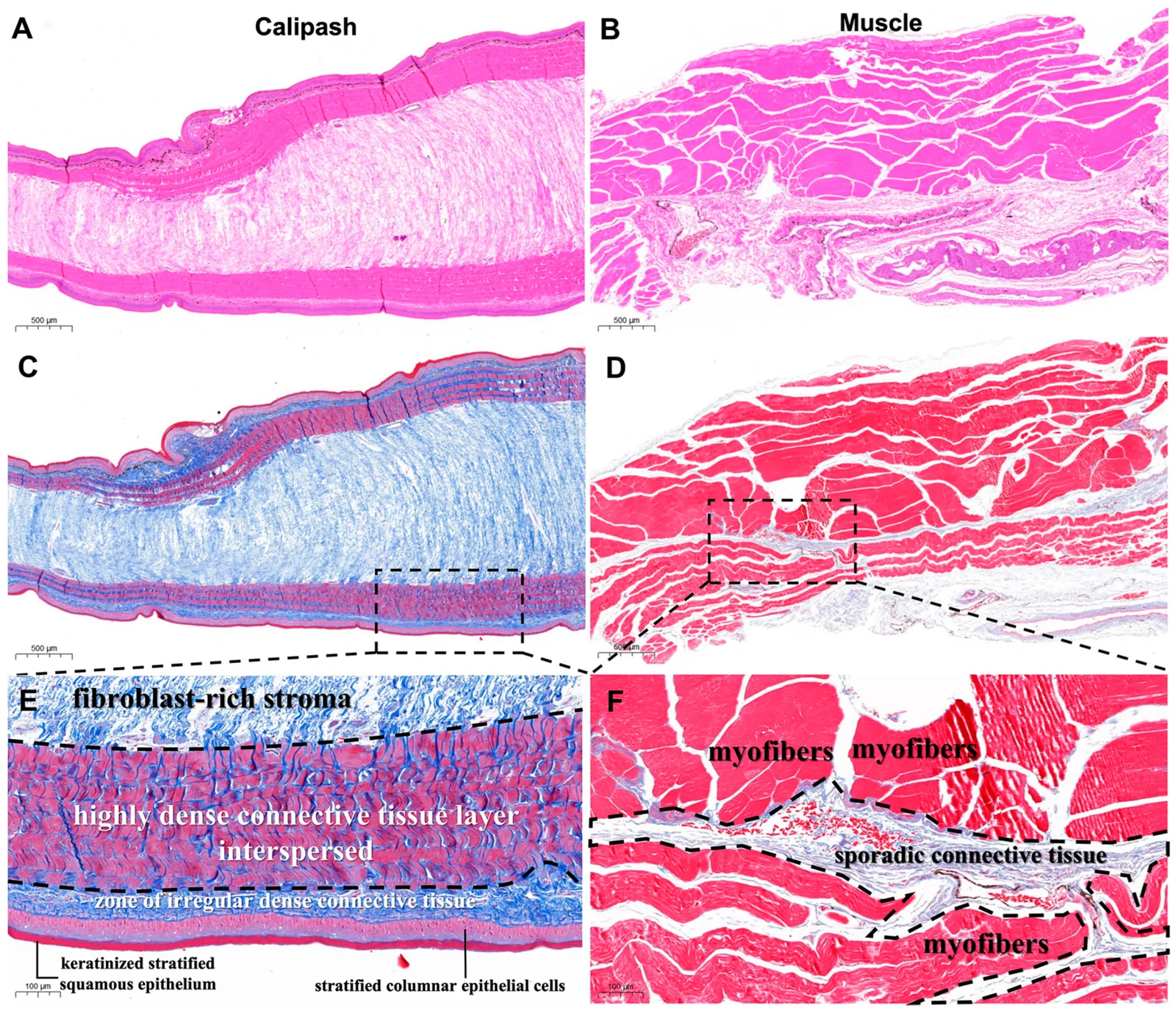
Comparative histological micrographs of the calipash and muscle tissues in *P. sinensis*. (**A**,**B**) Representative H&E staining of the calipash (**A**) and muscle (**B**). (**C**,**D**) Corresponding low-magnification Masson’s trichrome staining of the calipash (**C**) and muscle (**D**). (**E**,**F**) High-magnification views of the Masson’s trichrome staining corresponding to the boxed regions in (**C**,**D**). Scale bars are as indicated in the respective images.

To elucidate the structural basis underlying the distinct textural properties of the calipash and muscle, a comparative histological analysis was performed. HE staining showed that the calipash exhibited a highly dense, strongly eosinophilic ECM beneath the outermost keratinized epithelium (in Figure 1A). In contrast, muscle displayed a highly compartmentalized structure of myofiber bundles (Figure 1B). Masson’s staining revealed that the calipash exhibited a multilayered architecture with extensive collagen-rich connective tissue (Figure 1C), whereas muscle was dominated by distinct red-stained myofiber bundles separated by interstitial spaces, with blue collagen signals largely confined to the limited mesenchymal connective tissue (Figure 1D).

High-magnification views further resolved the multilayered architecture of calipash (Figure 1E). From the surface inward, it consists of a keratinized stratified squamous epithelium (dark red), followed by a stratified columnar epithelial cell layer (red), a zone of irregular dense connective tissue (blue), a highly dense connective tissue layer containing blue collagen fibers interweaved with prominent red-stained fiber bundles, and an innermost fibroblast-rich stroma (blue) (Figure 1E). In contrast, muscle exhibited a uniform arrangement of myofibers, containing only sporadic loose connective tissue networks (Figure 1F).

### 3.2. Comparative Analysis of Collagen Content and Distribution Between Calipash and Muscle

**Figure 2:**
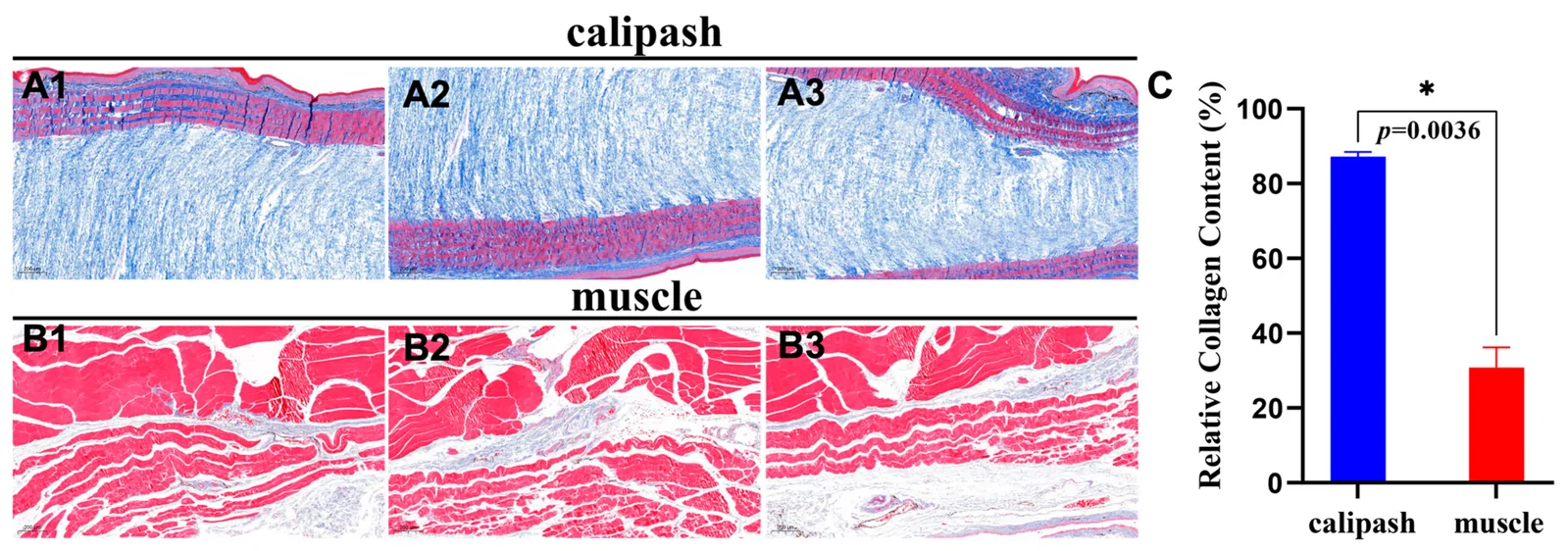
Quantitative analysis of collagen abundance in the calipash and muscle. (**A**,**B**) Representative Masson’s trichrome-stained sections utilized for collagen quantification in calipash (**A**) and muscle (**B**). (**A1**–**A3**) The first/second/third microscopic field in calipash; (**B1**–**B3**) The first/second/third microscopic field in muscle. The microscopic images are representative fields from the same individual and were used for visualization only. (**C**) Quantitative comparison of the collagen area fraction between the calipash and muscle tissues. Data are presented as mean ± SD (*n* = 3) calculated from three independent microscopic fields. * *p* < 0.05 indicates significant differences.

Based on these morphological observations, we hypothesized that collagen abundance differed significantly between calipash and muscle. Visual inspection revealed a larger area of blue-stained collagen signals in the calipash compared with the muscle (Figure 2A,B). Quantitative analysis was performed on three individual animals (*n* = 3) per tissue, with three microscopic fields (Figure 2(A1–A3,B1–B3)) analyzed per animal. Consistent with the histological observations, the collagen-positive area fraction was significantly higher in the calipash than in muscle (Figure 2C).

**Figure 3:**
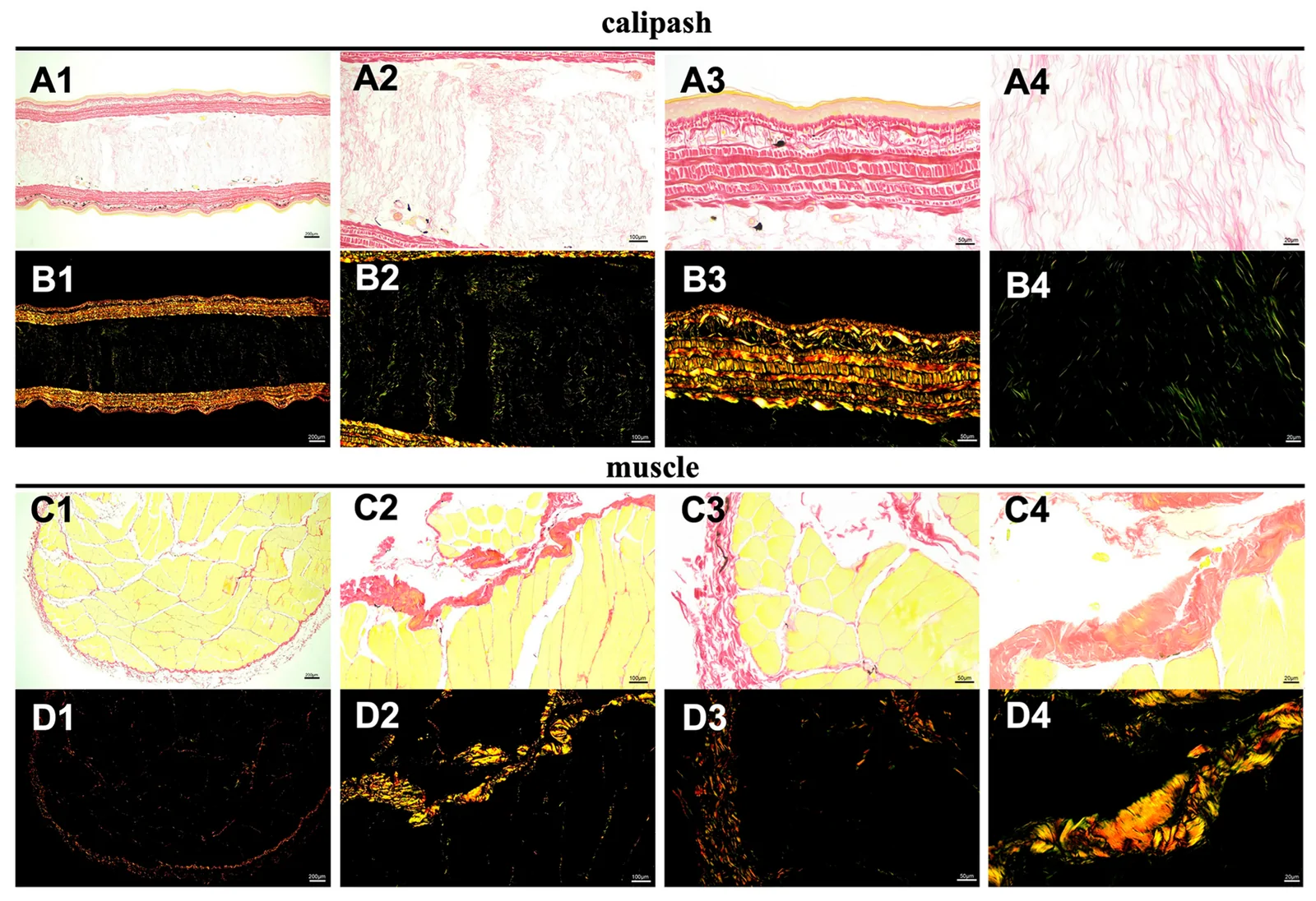
Comparative Picro Sirius Red staining of collagen subtype composition and spatial organization. (**A1**–**A4**) Representative brightfield microscopy images of the calipash acquired at increasing magnification. Scale bars: 200 μm (**A1**), 100 μm (**A2**), 50 μm (**A3**) and 20 μm (**A4**). (**B1**–**B4**) Representative polarized light microscopy images of the calipash acquired at increasing magnification. Scale bars: 200 μm (**B1**), 100 μm (**B2**), 50 μm (**B3**) and 20 μm (**B4**). (**C1**–**C4**) Representative brightfield microscopy images of muscle acquired at increasing magnification. Scale bars: 200 μm (**C1**), 100 μm (**C2**), 50 μm (**C3**) and 20 μm (**C4**). (**D1**–**D4**) Representative polarized light microscopy images of muscle acquired at increasing magnification. Scale bars: 200 μm (**D1**), 100 μm (**D2**), 50 μm (**D3**) and 20 μm (**D4**). Yellow-orange birefringence indicates densely packed type I collagen, while green birefringence is indicative of type III collagen-rich regions. Scale bars are as indicated in the respective images.

Intriguingly, Masson’s trichrome staining revealed prominent red-stained fiber bundles within the dense connective tissue layer of calipash (Figure 1C,E), visually resembling the staining pattern of myofibers in the muscle (Figure 1D,F). To clarify the characteristics of these red bundles, Sirius Red staining combined with polarizing light microscopy was performed. Under bright-field microscopy, these bundles in the calipash were stained red (Figure 3(A1–A3)), whereas the myofibers in the muscle tissue remained unstained (Figure 3(C1–C3)), suggesting a fundamental compositional difference. Under polarized light, the red bundles in calipash exhibited intense yellow–orange birefringence (Figure 3(B3)), confirming their identity as collagen fibers rather than muscle fibers, which lacked any birefringent signals (Figure 3(D3)). Furthermore, the innermost loose connective tissue stroma exhibited a mixture of yellow and green signals, with the green birefringence being even more intense (Figure 3(B4)). In contrast, the interstitial loose connective tissue exhibited predominantly yellow–orange birefringence with fewer green signals in the muscle (Figure 3(D4)). Collectively, these findings not only confirm that the red bundles observed in Masson’s staining in the calipash are collagenous rather than muscular, but also delineate the differential collagen composition and distribution between the calipash and muscle.

**Figure 4:**
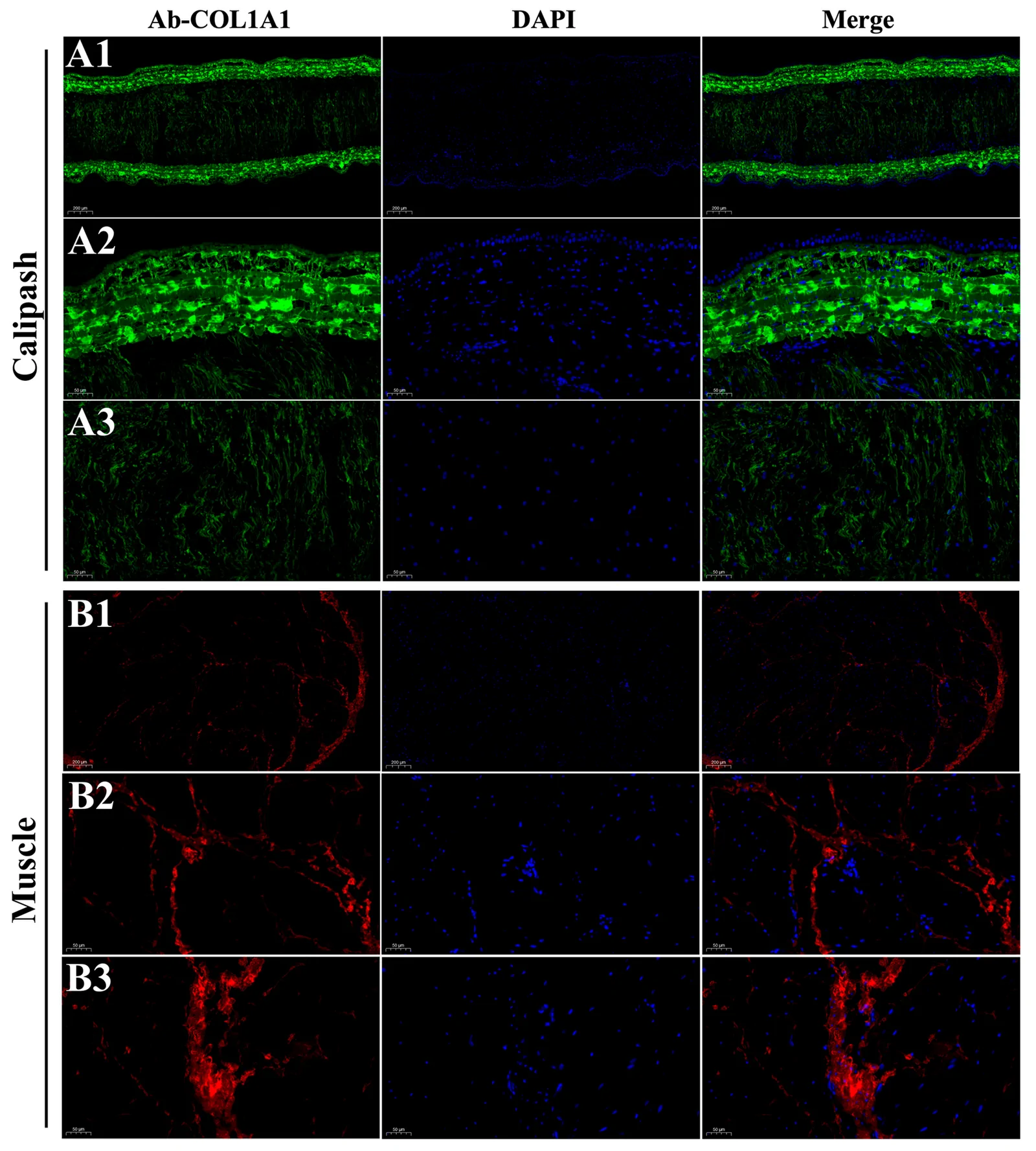
Comparative immunofluorescence localization of COL1A1 in calipash (**A1**–**A3**) and muscle (**B1**–**B3**) tissues. (**A1**) Representative immunofluorescence image showing the spatial distribution of COL1A1 (green) in calipash at low magnification; scale bar, 200 μm. (**A2**) Higher-magnification image of a representative field of view in calipash; scale bar, 50 μm. (**A3**) Higher-magnification image of a second, independent field of view in calipash; scale bar, 50 μm. (**B1**) Spatial distribution of COL1A1 (red) in muscle tissue at low magnification; scale bar, 200 μm. (**B2**) Higher-magnification image of a representative field of view in muscle; scale bar, 50 μm. (**B3**) Higher-magnification image of a second, independent field of view in muscle; scale bar, 50 μm. Nuclei were counterstained with DAPI (blue). Scale bars are as indicated in the respective images.

Additionally, to explicitly validate the spatial distribution of type I collagen, COL1A1 immunofluorescence (IF) staining was performed. The COL1A1 fluorescent signal was widely distributed throughout the calipash, except for the outermost epithelial cell layer (Figure 4A). Specifically, an intensely strong green, fluorescent signal was detected in the connective tissue immediately beneath the epithelium (Figure 4(A2)). In the innermost layer, COL1A1 signals exhibited a highly organized, bundle-like arrangement (Figure 4(A3)). In muscle, COL1A1 signals were mainly located in the interstitial space between muscle fibers (Figure 4B). Collectively, these observations provide complementary histological and protein-level evidence for distinct collagen distributions between calipash and muscle.

### 3.3. Global Transcriptomic Profiling and Tissue-Specific Functional Divergence

**Figure 5:**
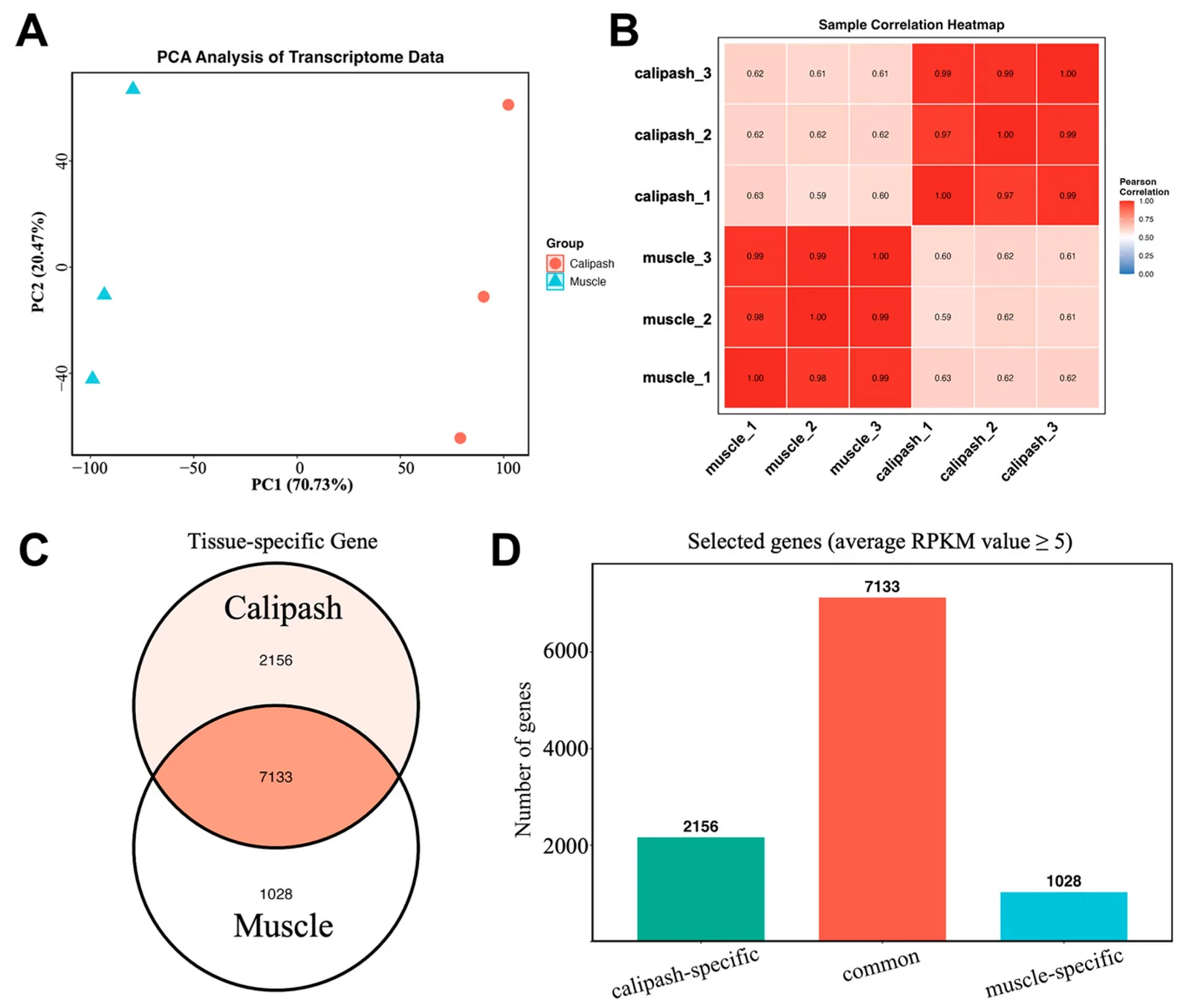
Clustering analysis of transcriptome data and identification of tissue-specific genes. (**A**) Principal component analysis (PCA); (**B**) Pearson correlation analysis of transcriptomic data of calipash and muscle. Schematic diagram ((**C**), Venn chart; (**D**), bar chart) of calipash-specific and muscle-specific expressed genes. Genes with a mean FPKM ≥ 5 across biological replicates were considered reliably expressed. Genes with average FPKM ≥ 5 in one tissue and FPKM < 5 in the other tissue across all three replicates were considered tissue-specific.

To further investigate the molecular basis underlying the observed histological differences between the calipash and muscle, comparative transcriptome analysis was performed. Principal component analysis (PCA) revealed a clear separation between the calipash and muscle along the first principal component (PC1), with no overlap between biological replicates of the two tissues (Figure 5A). Pearson correlation analysis further confirmed high intra-group consistency and pronounced inter-group differences (Figure 5B), indicating distinct transcriptional profiles between the calipash and muscle.

**Figure 6:**
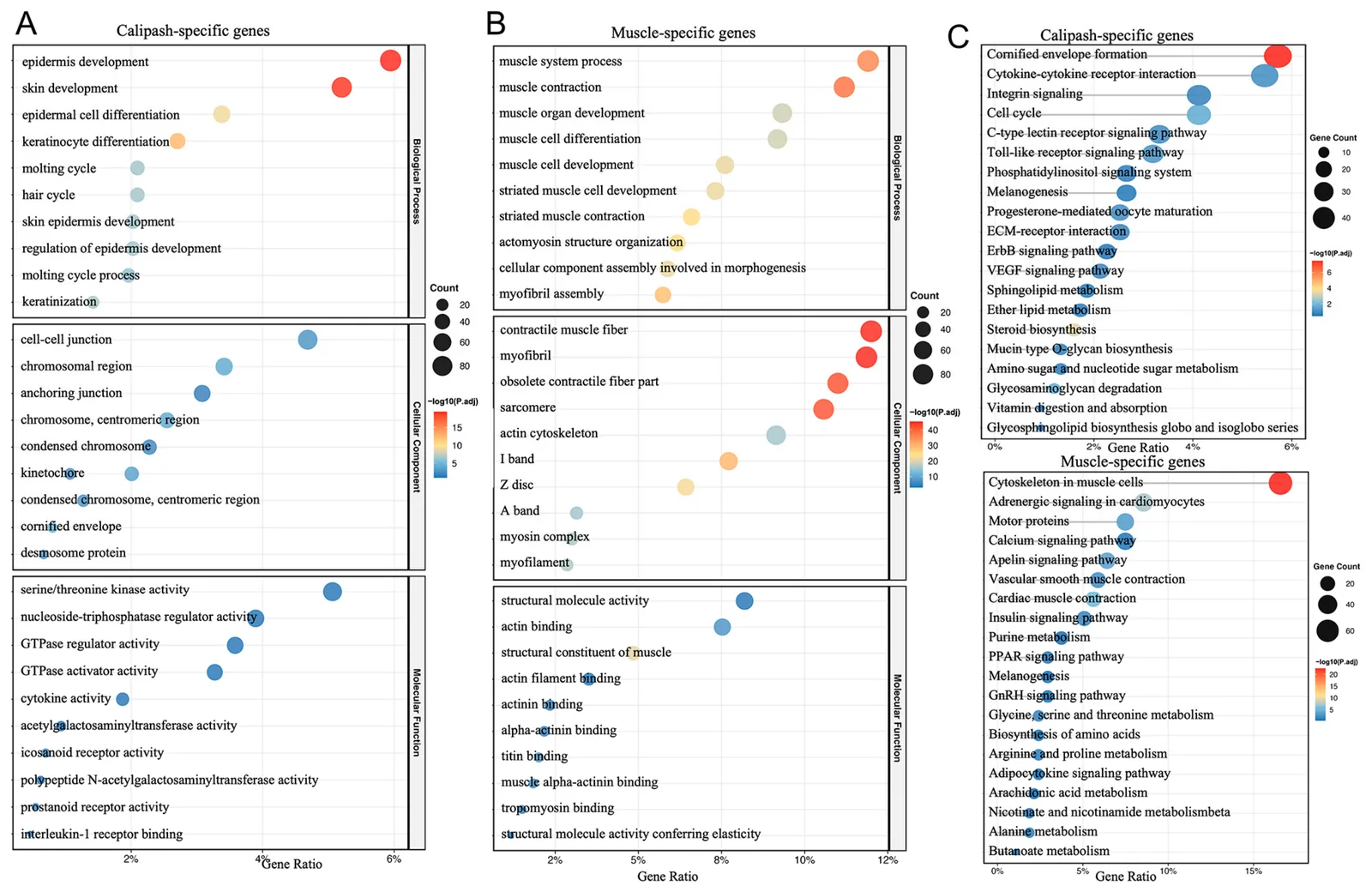
Enrichment analysis of calipash-specific genes and muscle-specific genes. GO enrichment analysis of calipash-specific genes (**A**) and muscle-specific genes (**B**). KEGG pathway enrichment analysis of calipash-specific genes and muscle-specific genes (**C**).

To systematically elucidate the functional divergence between the two tissues, Gene Ontology (GO) and KEGG pathway enrichment analyses were conducted. For calipash-specific genes, 2156 calipash-specific and 1028 muscle-specific genes were identified (Figure 5C,D). GO enrichment of calipash-specific genes was predominantly driven by biological processes (BP) related to epidermis development, keratinocyte differentiation and the molting cycle. Cellular component (CC) and molecular function (MF) analyses further highlighted terms critical for tissue integrity and signaling, such as anchoring junction, cornified envelope and GTPase regulator activity (Figure 6A). KEGG pathway analysis revealed a significant enrichment in ECM-receptor interaction and immune-related pathways, including Toll-like and C-type lectin receptor signaling), as well as barrier-related metabolic processes (e.g., Sphingolipid metabolism (Figure 6C). These enrichment patterns were consistent with the epidermal, ECM-related, and signaling characteristics of the calipash.

In contrast, the muscle-specific genes exhibited highly conserved functional roles centered on mechanical output. These genes were overwhelmingly enriched in contraction-related GO terms across all categories, including muscle contraction and myofibril assembly (BP), sarcomere and Z disk (CC), as well as actin binding and structural constituent of muscle (MF) (Figure 6B). KEGG analysis further corroborated this functional specialization, with the top pathways predominantly clustering into contractile execution (e.g., calcium signaling pathway and cardiac muscle contraction) and diverse cellular energy metabolisms (e.g., PPAR signaling pathway and purine metabolism) (Figure 6C). Together, these results confirm that the muscle transcriptome is highly specialized for structural assembly, energy metabolism, and contractile execution.

### 3.4. Differential Expression Profiling of Collagen Gene Families

Differential expression analysis was performed with the calipash as the reference group to identify transcriptional differences between the muscle and calipash. A total of 6916 differentially expressed genes (3453 down and 3463 up DEGs) were identified (Figure 6A).

**Figure 7:**
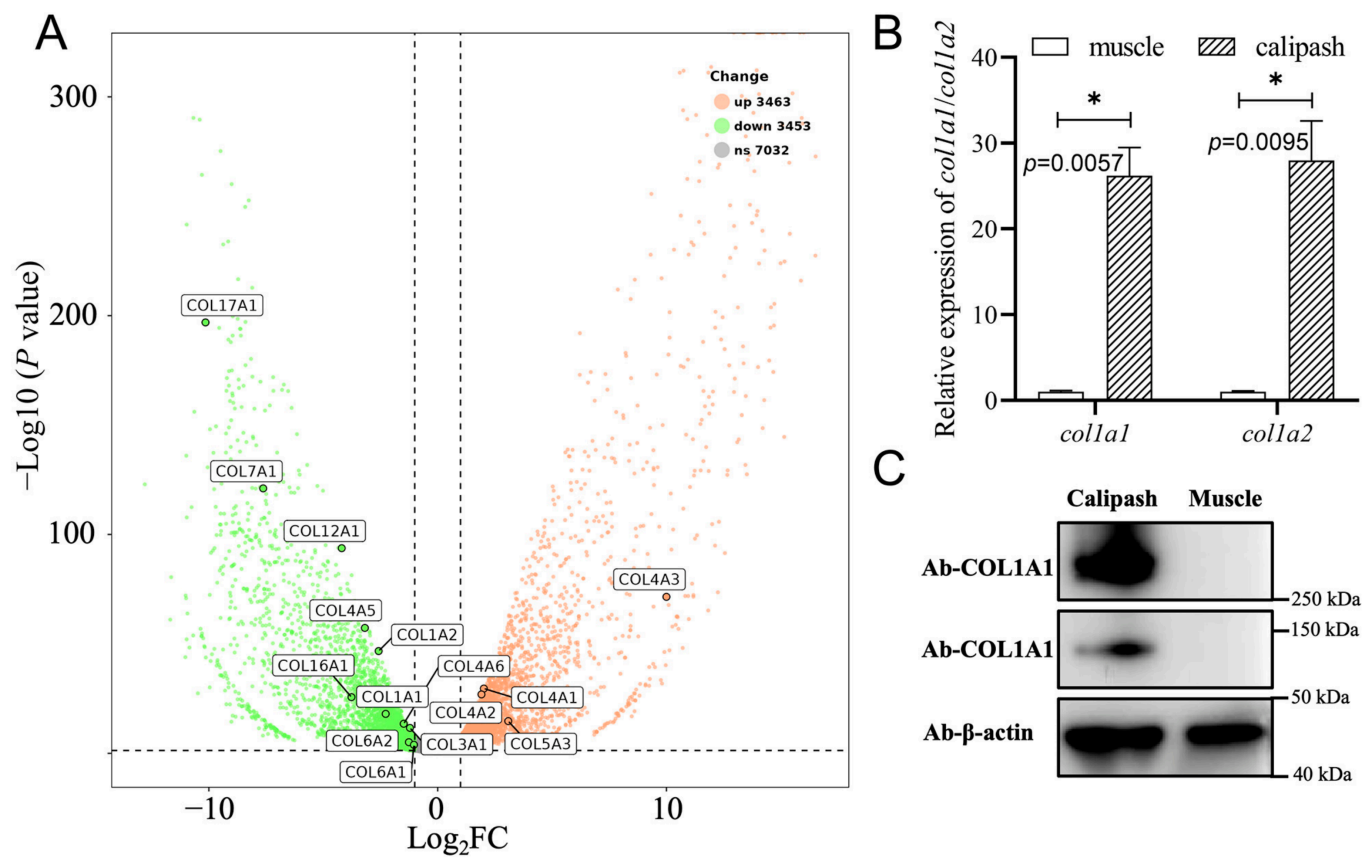
Collagen gene expression profiling, transcriptional validation, and protein-level confirmation in calipash and muscle of *P. sinensis*. (**A**) Volcano plot of collagen-related DEGs. Each point represents a gene, with significantly upregulated (green) and downregulated (orange) genes in calipash defined by |log_2_ fold change| ≥ 1 and FDR < 0.05. The *x*-axis represents log_2_ fold change, and the *y*-axis represents log_10_ adjusted *p* value. Collagen-related genes are highlighted, including *col1a1*, *col1a2*, *col3a1*, *col6a1*, *col6a2*, *col4a6*, *col4a5*, *col5a3*, *col7a1*, *col12a1*, *col16a1*, and *col17a1*. (**B**) Targeted qPCR validation of *col1a1* and *col1a2* expression in calipash and muscle. Data are presented as mean ± SD (*n* = 3). Asterisks indicate significant differences between calipash and muscle (*p* < 0.05). (**C**) Western blot analysis of COL1A1 protein in calipash and muscle. β-actin was used as the loading control.

To further explore the differences in collagen-related structures between calipash and muscle, we systematically analyzed the expression profiles of collagen-encoding genes identified among the 6916 DEGs (Figure 7A and Table 1). Fibrillar collagen genes, particularly collagen alpha-2(I) (*col1a1*) and collagen alpha-1(I) (*col1a2*), exhibited the highest transcription level among all collagen genes in both tissues (Table 1). Notably, the transcript abundance of *col1a1/2* in the calipash was approximately 6- to 8-fold higher than that in the muscle. Collagen alpha-1(III) (*col3a1*) ranked second in transcript abundance and also exhibited markedly higher expression in the calipash. This transcriptional pattern was consistent with the polarized light microscopy observations, in which the innermost stromal layer of the calipash exhibited mixed yellow and green birefringent signals (Figure 3), supporting the presence of both type I and type III collagen. Other collagen subtypes, including collagen alpha-1(VI) (*col6a1*), collagen alpha-2(VI) (*col6a2*), collagen alpha-6(IV) (*col4a6*), collagen alpha-5(IV) (*col4a5*), collagen alpha-3(V) (*col5a3*),collagen alpha-1(VII) (col7a1), collagen alpha-1(XII) (*col12a1*), and collagen alpha-1(XVI) (*col16a1*), were also detected at lower transcript levels in both calipash and muscle (Table 1), indicating a diverse ECM composition.

### 3.5. Targeted Validation of Type I Collagen at Transcript and Protein Levels

The relative mRNA expression of *col1a1/2* in the calipash was significantly higher than in muscle, corroborating the transcriptomic quantification. Western blot analysis further confirmed its protein abundance. In the muscle, no specific COL1A1 band was detected (Figure 7C). In the calipash, two immunoreactive bands were observed: a monomeric α1(I) chain at approximately 150 kDa and a more intense band above 250 kDa (Figure 7C). The latter may represent a γ-trimer or other higher-order collagen form [34,35], consistent with enhanced collagen assembly and maturation in calipash.

### 3.6. Validation of RNA-seq Data by RT-qPCR

**Figure 8:**
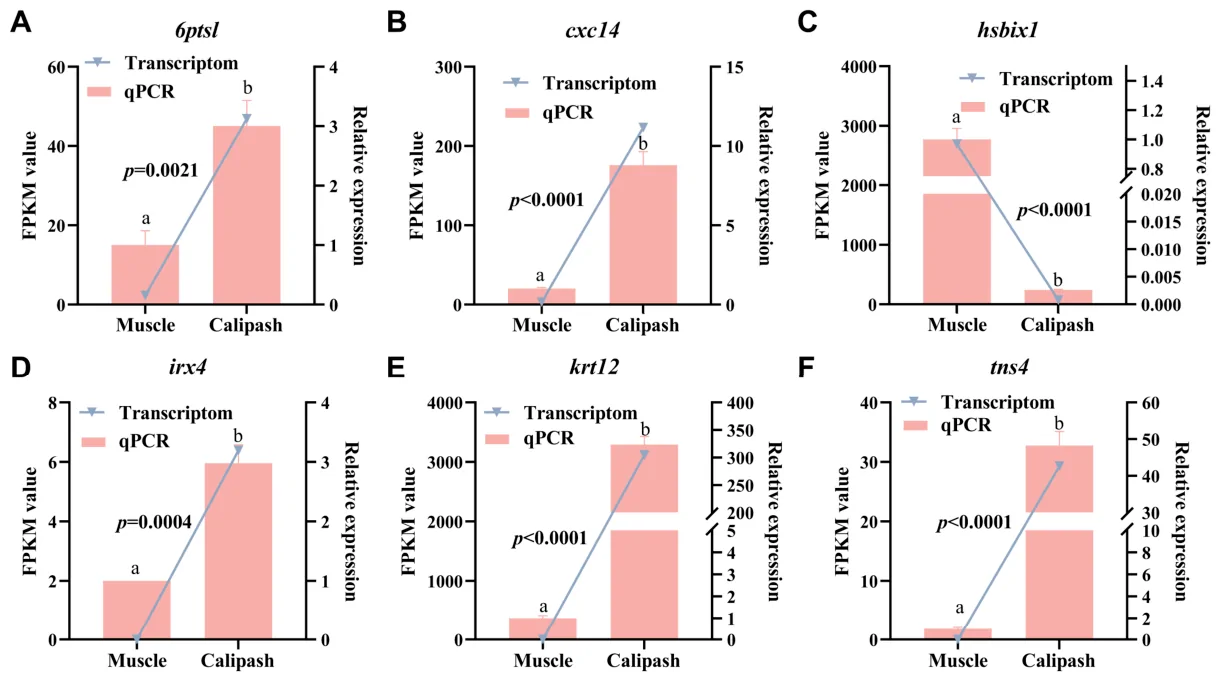
qPCR validation in calipash and muscle tissues. The genes are 6-pyruvoyl tetrahydrobiopterin synthase-like (*6ptsl*, (**A**)), C-X-C motif chemokine ligand 14 (*cxc14*, (**B**)), (hemoglobin subunit beta-like (*hsbix1*, (**C**)), iroquois homeobox 4 (*irx4*, (**D**)), keratin type I cytoskeletal 12 (*krt12*, (**E**)), *tensin 4* (*tns4*, (**F**)). Significant differences (*p* < 0.05) between groups are indicated by distinct lowercase letters.

To validate the reliability and reproducibility of the RNA-seq expression profiles, six DEGs were randomly selected for qPCR analysis. As shown in Figure 8, the relative expression trends determined by qPCR were highly consistent with the FPKM values obtained from the RNA-seq data, confirming the accuracy of our transcriptomic analysis.

## 4. Discussion

Calipash and muscle exhibit fundamentally distinct biological organizations. Calipash-specific genes were predominantly enriched in epidermal development, keratinocyte differentiation, the molting cycle, sphingolipid metabolism, and immune-related pathways, consistent with transcriptional signatures reported in vertebrate integumentary tissues [8,36,37,38]. In contrast, muscle-specific genes were mainly associated with oxidative phosphorylation, mitochondrial function, and myofibril assembly, consistent with the conserved molecular characteristics of skeletal muscle [39,40,41,42]. These transcriptional profiles were consistent with the distinct histological architectures of the two tissues (Figure 1).

Strong birefringent signals in the connective tissue layers revealed by Sirius Red staining combined with polarizing light microscopy [43,44] indicated abundant and highly organized collagen deposition in the calipash, consistent with previous observations in *P. sinensis* [5]. Although we acknowledge that birefringence color alone cannot definitively distinguish collagen types, the integration of polarizing light microscopy (Figure 3) with COL1A1 immunofluorescence (Figure 4) and transcriptomic profiling of *col1a1/2* and *col3a1* (Table 1) provides evidence for the differential distribution of type I and type III collagens. *Col1a1/2*, exhibited markedly higher expression in the calipash than in the muscle, supporting the prominent collagen deposition observed histologically. These differences in collagen abundance and composition may contribute to their distinct tissue architectures and, consequently, their characteristic textural properties.

The collagen architecture of the calipash differs markedly from that of the muscle interstitium, which contains only sparse collagen deposits primarily serving to separate myofibers [45,46]. It also differs from the collagen organization characteristic of the mammalian dermis [47,48,49,50,51]. In mammalian skin, dermal collagen is generally organized into papillary and reticular layers, with differences in fiber thickness, orientation, and extracellular matrix composition across these regions [47,48,49,50,51]. Although the calipash shares features of a skin-associated connective tissue, its collagen organization and spatial distribution may differ substantially from those of the mammalian dermis. These distinctive structural features may contribute to the specialized textural properties of the calipash.

To our knowledge, this study provides the first biochemical evidence by Western blotting beyond transcriptional and histological evidence for collagen abundance. Specifically, a dominant high-molecular-weight band (>250 kDa) was observed in the calipash in addition to the monomeric α1(I) chain (~150 kDa). Based on the characteristic SDS-PAGE migration pattern of type I collagen, this band is consistent with the covalently cross-linked γ-trimer (composed of two α1(I) chains and one α2(I) chain). The predominance of the high-molecular-weight collagen species over the monomeric α1(I) chain may indicate a greater degree of collagen assembly and maturation in the calipash. This interpretation is further supported by densely organized collagen fibers in the inner layer of the calipash (Figure 2, Figure 3 and Figure 4). Although the high-molecular-weight band cannot be definitively assigned to a γ-trimer based on the present evidence, its presence together with the pronounced organization of type I collagen fibers observed histologically is consistent with enhanced collagen assembly and maturation in the calipash [52]. Such distinctive collagen organization likely reflects the biomechanical demands of the semi-aquatic lifestyle. Our findings are broadly consistent with previous molecular studies of *P. sinensis* showing collagen-related changes during embryonic calipash development [10] and age-dependent muscle characteristics [11]. The formation and stabilization of the higher-order collagen species observed here may involve lysyl oxidase (LOX) and other collagen cross-linking enzymes [53], although these mechanisms were not directly examined in the present study and warrant further investigation.

Interestingly, although Masson’s trichrome staining is commonly used to distinguish collagen-rich regions [22,54], we observed an unusual staining pattern in the calipash. Prominent fiber bundles within the dense connective tissue layer retained red rather than the expected blue staining typically associated with collagen fibers. This pattern may reflect differences in the organization or packing of collagen fibers that affect dye accessibility. Importantly, intense yellow–orange birefringence observed with polarizing light microscopy confirmed that these red bundles are indeed collagen fibers rather than myofibers. These observations indicate a dense and highly organized collagen architecture in the calipash. Such organization may contribute to its distinct structural and textural properties, including firmness, elasticity, and the characteristic gelatinous texture after cooking, although this potential relationship requires direct biomechanical validation.

The present findings also have several limitations. The proposed association between collagen packing and the atypical Masson’s staining pattern remains hypothetical and would require ultrastructural or physicochemical analyses to determine whether collagen density or organization directly affects dye accessibility. Similarly, the high-molecular-weight collagen species observed by Western blotting may represent a γ-trimer or other higher-order collagen form, but its identity and cross-linking status cannot be definitively established from the present data. Additional approaches, such as electron microscopy, analysis under reducing conditions, or mass spectrometric identification, would be valuable for further characterizing collagen assembly and maturation in the calipash.

## 5. Conclusions

In conclusion, this study provides a histological, protein-level, and transcriptomic characterization of the calipash and muscle in *P. sinensis*, revealing distinct tissue architectures and molecular profiles. The calipash exhibited epidermal differentiation and a compact, highly organized ECM, with prominent type I collagen deposition. A high-molecular-weight collagen species was also detected in the calipash, which may represent a γ-trimer or other higher-order collagen form and is consistent with enhanced collagen assembly and maturation. In contrast, the muscle exhibited a transcriptional profile characterized by oxidative phosphorylation, mitochondrial function, and myofibril assembly, consistent with its contractile function. These findings highlight marked differences in collagen organization and molecular characteristics between the calipash and muscle and provide a basis for understanding their distinct structural and textural properties.

## Figures and Tables

**Table 1:** DEGs related to collagen in the muscle and calipash of *P. sinensis*.

Gene Name	FPKM						Log_2_FC
	Muscle1	Muscle2	Muscle3	Calipash1	Calipash2	Calipash3	
*col1a2*	243.46	224.04	238.86	1971.93	1763.1	1839.12	−2.57905287
*col1a1*	63.18	97.88	86.68	407.82	647.69	507.58	−2.27124560
*col3a1*	95.43	95.22	98.36	306.16	286.89	288.66	−1.208861171
*col6a2*	52	92.96	79.91	162.97	295.48	242.91	−1.25666288
*col6a1*	49.03	83.71	68.24	129.36	228.06	180.97	−1.03740672
*col17a1*	0.12	0.04	0.08	115.53	135.88	135.65	−10.1461673
*col12a1*	3.4	2.69	3.06	83.28	62.65	76.04	−4.19258523
*col4a5*	3.85	3.37	3.48	42.03	41.11	44.93	−3.181810744
*col7a1*	0.14	0.14	0.13	28.91	43.58	34.54	−7.62492522
*col16a1*	1.04	2.17	1.7	18.72	40.2	27.74	−3.76776665
*col4a6*	4.62	4.96	5.17	17.2	18.64	18.42	−1.48279866
*col5a3*	8.33	15.95	12.31	0.94	2.87	1.78	3.08462533

Note: Log_2_FC = log2(FPKM_muscle/FPKM_calipash); negative values indicate higher expression in calipash. Gene symbols are standardized.

## Data Availability

All raw sequencing data generated in this study have been deposited in the NCBI BioProject database with accession numbers PRJNA1332075.

## Appendix A

**Table A1:** All the primers listed in this study.

Primer Name	Primer Sequence 5′–3′	Usage
*β-actin*	F:GAGACCTGACAGACTACCT R:AGGATGATGAAGCAGCAGT	qPCR
*col1a1*	F:GCAAAGACGGCCCAAGAGGT R:CAGGTTGGCCGTCAGAACCA	
*col1a2*	F:CAGGCACTGACGGTAACAAG R:GGGATTCCACCAGAACCAGA	
*6ptsl*	F:CTTCGACAGCGACTGTGCTC R:GTCCGTGACTGTGGTTGCAT	
*cxc14*	F:CCCTGACAGCAGCTTTACTTCT R:CTGAACCGCGACTGCATAGT	
*hsbix1*	F:CTTCGGGAACCTCTCGAACC R:CGTTGCCCAGGAGCTTGAAA	
*Krt12*	F:GCTTTGGTGGAGCTGATTGC R:TGCAATGACAGCCTAACCCA	
*irx4*	F:TTGCTGTGAGTCTAGTGGCA R:CCCTGGGTACTGGTGTAAGG	
*tns4*	F:ACGACGACTCGGACTCTTAC R:GCCATGGAGGTAACGAGAGA	
